# Global health and global justice

**DOI:** 10.1186/1471-2458-14-S1-I1

**Published:** 2014-01-29

**Authors:** Norman Daniels

**Affiliations:** 1 Harvard University, Cambridge, MA, USA

## 

Most people are concerned about international health inequalities and find them unjust. At the same time, we lack global institutions that can effectively act to reduce these health inequalities, which have various sources. Accordingly, the concern about health inequalities does not by itself support some one of the competing theories of global justice. Yet, many people believe that specific problems increase injustice globally and might be addressed by various measures: the brain drain of health workers from poorer to richer countries, the lack of development and dissemination of drugs that address the global burden of disease, the high rates of mortality (and morbidity) that reflect obvious weaknesses in health systems (e.g. maternal mortality rates), the lack of universal coverage for important health services, the lack of effective global public goods, such as better surveillance systems. I recommend an approach to developing an account of global justice that builds on promising solutions to such specific issues, where these solutions build sustainable institutions that address the problems.

